# Credit assignment in hierarchical option transfer

**Published:** 2022-07

**Authors:** Jing-Jing Li, Liyu Xia, Flora Dong, Anne G.E. Collins

**Affiliations:** Helen Wills Neuroscience Institute, University of California, Berkeley; Department of Mathematics, University of California, Berkeley; Department of Psychology, University of California, Berkeley; Department of Psychology, Helen Wills Neuroscience Institute, University of California, Berkeley

**Keywords:** hierarchical reinforcement learning, the options framework, transfer learning, credit assignment

## Abstract

Humans have the exceptional ability to efficiently structure past knowledge during learning to enable fast generalization. Xia and Collins (2021) evaluated this ability in a hierarchically structured, sequential decision-making task, where participants could build “options” (strategy “chunks”) at multiple levels of temporal and state abstraction. A quantitative model, the Option Model, captured the transfer effects observed in human participants, suggesting that humans create and compose hierarchical options and use them to explore novel contexts. However, it is not well understood how learning in a new context is attributed to new and old options (i.e., the credit assignment problem). In a new context with new contingencies, where participants can recompose some aspects of previously learned options, do they reliably create new options or overwrite existing ones? Does the credit assignment depend on how similar the new option is to an old one? In our experiment, two groups of participants (n=124 and n=104) learned hierarchically structured options, experienced different amounts of negative transfer in a new option context, and were subsequently tested on the previously learned options. Behavioral analysis showed that old options were successfully reused without interference, and new options were appropriately created and credited. This credit assignment did not depend on how similar the new option was to the old option, showing great flexibility and precision in human hierarchical learning. These behavioral results were captured by the Option Model, providing further evidence for option learning and transfer in humans.

## Introduction

Via reinforcement learning (RL), biological and autonomous agents can learn to take actions in an environment to maximize future cumulative reward (Sutton & Barto, 2018; Collins, 2019). In many real-world applications of artificial RL, it is beneficial, and sometimes necessary, for the agent to generalize or transfer past knowledge to solve new tasks (Wang et al., 2020). However, compared to humans, artificial agents are much less data-efficient transfer learners: they often require millions of training examples to adapt to changed reward contingencies in complex environments (Neftci & Averbeck, 2019). This discrepancy in learning abilities between natural and artificial intelligence leads to the question: how do humans structure past knowledge to generalize in new contexts (Collins, 2018)?

To answer this question, it is essential to understand how humans represent and compose knowledge. A common theme in behavior and cognitive representation is hierarchical organization, in which representation is decomposed into a hierarchy of substructures. Human action has long been recognized as hierarchically structured with nested subroutines rather than flat (i.e., non-hierarchical) stimulus-response associations (Jeffress, 1951; Miller, Galanter, & Pribram, 1960). This hierarchical structure is reflected in the functional organization of the prefrontal cortex (Badre & D’Esposito, 2007; Koechlin & Jubault, 2006; Koechlin, Ody, & Kouneiher, 2003). More recent works have sought to explain the crucial role hierarchy plays in decision-making (Balleine, Dezfouli, Ito, & Doya, 2015; Dezfouli & Balleine, 2013) and learning (Collins & Frank, 2013; Eckstein & Collins, 2020). With hierarchically represented knowledge, humans can compose lower-level chunks in novel ways to solve new tasks (Lake, Ullman, Tenenbaum, & Gershman, 2017; Xia & Collins, 2021).

To understand the computational mechanisms of hierarchically organized behavior, cognitive scientists have developed hierarchical models of human behavior inspired by computational RL (Botvinick, 2008), especially the options framework (Sutton, Precup, & Singh, 1999). Extending classic RL, the options framework generalizes single-step, primitive actions to include temporally extended action sequences (i.e., options, or strategy ”chunks”), forming hierarchies in time. This framework has drawn attention in cognitive science due to its resemblance to psychological accounts of behavior and benefits over traditional, flat RL, including better scalability, more efficient exploration, and longer-term planning (Botvinick, Niv, & Barto, 2009; Botvinick & Weinstein, 2014). Under the options framework, each option is characterized by a set of states where it is initiated, a termination condition, and an option-specific policy that maps states to actions or options. Thus, selecting an option in one decision step may trigger a series of decisions defined by the option.

Recent work has shown that the options framework is not only plausible, but highly successful, as a model of human learning and decision-making. In a number of experiments, human participants identified meaningful subgoals (i.e., secondary goals that were not directly rewarded) (Diuk, Schapiro, et al., 2013; Schapiro, Rogers, Cordova, Turk-Browne, & Botvinick, 2013) and used reward prediction error to track subtask progression (Diuk, Tsai, Wallis, Botvinick, & Niv, 2013; Ribas-Fernandes, Shahnazian, Holroyd, & Botvinick, 2019; Ribas-Fernandes et al., 2011). Using a behavioral task with contingencies forming hierarchies over time and states, Xia and Collins (2021) extended the options framework to capture multiple types of generalizable hierarchical chunks. Their results showed that humans create and compose hierarchical options, and use them to explore novel contexts, consequently transferring past knowledge.

However, there has been a lack of data and accounts for how new and old options are credited in new contexts where options may be transferred or recomposed (i.e., how much of the learning can be attributed to new vs. old options). Here, using modified versions of the experimental paradigm introduced by Xia and Collins (2021), we tested human participants’ performance in learning and transferring options defined by hierarchical contingencies. We aimed to answer two questions: a) after learning a set of options, when some contingencies of these options are updated, do human participants create new options or overwrite existing ones? b) Does the credit assignment of new and old options depend on how similar the updated contingencies are to those learned originally?

## Methods

### Participants

All experiments were approved by the Institutional Review Board at University of California, Berkeley. 412 undergraduates completed the experiment and received course credits for participation. Recruitment was restricted to native or fluent English speakers above 18 with no significant history of brain injury, mental/psychiatric illness, and alcohol or drug abuse.

### Design and procedure

**Figure 1:**
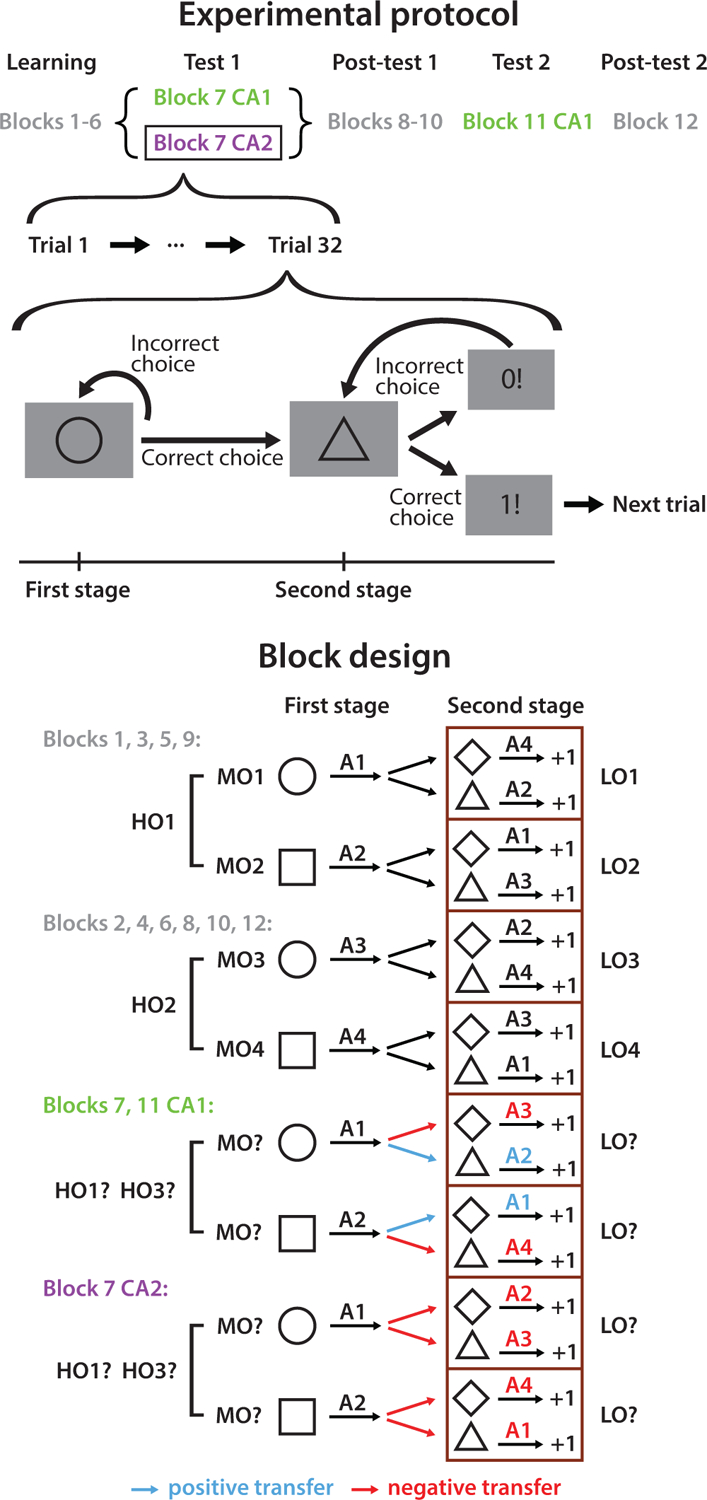
Experimental design. In each stage of a trial, participants learned the correct response to each stimulus among four keys (A1–A4): they must press the correct key before transitioning to the next stage or trial. The correct stimulus-response pairings were hierarchically designed and changed across blocks such that we could test whether previously learned options may be transferred to facilitate new learning.

We used a sequential decision-making task with a hidden hierarchical structure that participants could discover via trial and error (Fig. 1). The hierarchical structure included temporal dependencies that allowed us to test for options: the correct choice for a stimulus depended on previous choices in the current context. Participants had the opportunity to learn options at three levels of abstraction: high, medium, and low-level options (HO, MO, and LO). Across blocks, the contingencies changed to enable learning of two high-level options (HO_1_ and HO_2_), and then testing of new options (CA1 or CA2 for credit assignment), after which participants were re-tested on the old options in Post-test blocks. Each block consisted of either 60 trials (Blocks 1–2) or 32 trials (Blocks 3–12). In the 12-block versions of the experiment, participants skipped to the next block if they made less than 1.5 presses in the second stage of Block 1 or 2 for ten consecutive trials after the first 32 trials.

In each block, participants’ goal was to learn which sequence of key-press actions (e.g., A1 then A4) was the correct response to each of four possible sequences of shape stimuli (e.g., circle then diamond) based on deterministic, truthful feedback. Each trial included two stages: in the first stage, a circle or square was presented for 0.5 second and the participant pressed among four keys until reaching the correct response or ten presses in total, which allowed the trial to progress to the second stage. Participants had 2 seconds for each key press before they were notified of a timeout and asked to retry. The second stage proceeded in the same fashion, except that explicit feedback was given in response to each key press (1 for correct and 0 otherwise).

To ensure transfer effects were interpreted in participants who had learned effectively, only those who achieved above-chance performance in both stages at the end of Learning were included in our analysis. Test 1 tested participants’ responses to a new option context that could lead to different amounts of negative transfer of learned policies under HO_1_: CA1 and CA2 included two and four associations conflicting with HO_1_, respectively. Post-test 1, participants were retested on HO_1_ to help understand if the original HO_1_ policies were overwritten by the putative negative transfer in Test 1. Test 2 tested how well the options learned in Test 1 retained.

Each participant completed one of four versions of the task, which differed in the total number of blocks and the design of Test 1 (Table 1). Some participants completed the task in person, while others participated online. Each participant was equally likely to be tested on CA1 and CA2 in Test 1.

### Data analysis

We measured performance in a given stage of a trial using the number of key presses until the correct choice was reached. Fewer presses indicated better performance. Ceiling performance was 1 press and floor was 10 presses per stage per trial. Chance-level performance was 2.5 presses, assuming no wrong choice was repeated in the same stage of a trial. To evaluate transfer performance, we calculated the average number of presses in the second stage of the first ten trials of each block, before learning was saturated, and normalized it by the mean of Blocks 5 and 6. Statistical significance was tested using the Mann-Whitney U test for unpaired samples and Wilcoxon signed-rank test for statistics of the same population.

As we intended to study transfer effects, it is prerequisite that these contingencies were learned sufficiently by the end of the Learning blocks. As such, we only included participants who successfully learned the correct response sequences in the Learning phase (i.e., those who made an average of more than 2.5 presses per trial in either stage of the last 10 trials of either Block 5 or 6). 31% of 101 in-lab and 49% of 311 online participants were excluded, with 228 participants remaining in data analysis. While the exclusion rate is high, it is not rare for experiments with sparse reward outcomes and complex structures, particularly with online data. Furthermore, the exclusion allowed us to study the transfer of learned contingencies in the absence of added noise from participants who did not learn them well. However, we acknowledge that a non-trivial proportion of participants may not have successfully learned the correct strategy, the exclusion of whom might have created a skewed population.

### Computational modeling

Here, we evaluate the Option Model, which creates a new HO with new MOs and LOs in the beginning of each new block context (Xia & Collins, 2021). This mechanism allows it to update either existing or new options based on the context, leading to existing options being overwritten or new ones being learned during transfer.

We use a superscript 1 or 2 to indicate the first or second stage. In the first stage, the model selects an HO based on the probability *P*^1^ of each HO_*i*_ in the current context $c_{j}^{1}$, which encodes the current temporal (block) context, for 1 ≤ *i*, *j* ≤ *n*. Therefore, each block corresponds to a context in the first stage, and switching to the next block signals a transition into a new context. A new HO is created if and only if a new context is encountered, so the numbers of HOs and first-stage contexts are always the same. Upon encountering a new context $c_{n+1}^{1}$, the model creates a new HO_*n*+1_ whose probability of being sampled is

$$P^{1}\left({\text{HO}}_{n+1}|c_{n+1}^{1}\right)=\frac{\gamma^{1}}{Z^{1}}.$$

and the probability of reusing an existing HO_*i*_ for 1 ≤ *i* ≤ *n* is

$$P^{1}\left({\text{HO}}_{i}|c_{n+1}^{1}\right)=\frac{N_{i}^{1}}{Z^{1}},$$

where γ^1^ is the concentration parameter, $N_{i}^{1}=\sum_{k=1}^{n}P^{1}\left({\text{HO}}_{i}|c_{k}^{1}\right)$ is the cumulative probability of HO_*i*_ being chosen in all known contexts, and $Z^{1}=\gamma^{1}+\sum_{i=1}^{n}N_{i}^{1}$ is the normalization constant. The model implements Q-learning to track stimulus-action relationships. When HO_*n*+1_ is initialized, each stimulus-action pair is given an uninformative Q-value of $\frac{1}{4}$, as there are four possible actions in total. At decision-making time, the model chooses an action based on the softmax of the Q-values of the chosen HO for each candidate action $A_{j}^{1}$ with 1 ≤ *j* ≤ 4 that has not been tried in the current stage of the trial:

$$P\left(A_{j}^{1}|S^{1},\text{HO}\right)=\frac{\text{exp}\left(\beta^{1}\times Q_{\text{HO}}^{1}\left(S^{1},A_{j}^{1}\right)\right)}{\sum_{k=1}^{4}\text{exp}\left(\beta^{1}\times Q_{\text{HO}}^{1}\left(S^{1},A_{k}^{1}\right)\right)},$$

where *S*^1^ is the first-stage stimulus, and β^1^ is the softmax temperature parameter. Once an action *A*^1^ is chosen, a corresponding MO is activated for second-stage decision-making. Then the model observes the outcome of performing *A*^1^ and updates the probability of choosing every HO_*i*_ for 1 ≤ *i* ≤ *n*+1 using the Bayes’ Theorem:

$$P^{1}\left({\text{HO}}_{i}|c_{n+1}^{1}\right)\leftarrow \frac{P\left(r^{1}|S^{1},A^{1},{\text{HO}}_{i}\right)P\left({\text{HO}}_{i},c_{n+1}^{1}\right)}{\sum_{k=1}^{n+1}P\left(r^{1}|S^{1},A^{1},{\text{HO}}_{k}\right)P\left({\text{HO}}_{k},c_{n+1}^{1}\right)},$$

where the pseudo-reward *r*^1^ = 1 if *A*^1^ is correct and 0 if it is wrong. Note that $P\left(r^{1}|S^{1},A^{1},{\text{HO}}_{k}\right)=1-Q_{{\text{HO}}_{k}}^{1}\left(S^{1},A^{1}\right)$ if *r*^1^ = 0 and $Q_{{\text{HO}}_{k}}^{1}\left(S^{1},A^{1}\right)\;$ if *r*^1^ = 1. Then, the Q-value associated with *S*^1^ and *A*^1^ is updated by

$$Q_{\text{HO}}^{1}\left(S^{1},A^{1}\right)\leftarrow Q_{\text{HO}}^{1}\left(S^{1},A^{1}\right)+\alpha^{1}\times \left(r^{1}-Q_{\text{HO}}^{1}\left(S^{1},A^{1}\right)\right),$$

where α^1^ is the learning rate. At the end of the stage, the model forgets stimulus-action associations $\left(S_{i}^{1},A_{j}^{1}\right)\neq \left(S^{1},A^{1}\right)$ in the first stage with a forgetting rate of *f*^1^:

$$Q^{1}\left(S_{i}^{1},A_{j}^{1}\right)=\left(1-f^{1}\right)\times Q^{1}\left(S_{i}^{1},A_{j}^{1}\right)+f^{1}\times \frac{1}{4},$$

and it forgets all stimulus-action associations in the second stage with a forgetting rate of *f*^2^.

In the second stage, the model keeps track of the MO-specific probability $P_{\text{MO}}^{2}$ of choosing each LO in the given context $c_{j}^{2}$, which is characterized by the current block and first-stage stimulus. For each MO, $P_{\text{MO}}^{2}\left({\text{LO}}_{i}|c_{j}^{2}\right)$ is initialized and updated in the same way as $P^{1}\left({\text{HO}}_{i}|c_{j}^{1}\right)$ with a different concentration parameter γ^2^. Once an LO is chosen, action selection and Q-value updating are analogous to the first stage, with different softmax temperature parameter β^2^ and learning rate α^2^. Moreover, as participants quickly learned to avoid choosing the correct first-stage action in the second stage, a free meta-learning parameter *m* is added to account for this knowledge. After computing $P\left(A_{j}^{2}|S^{2}\right)$ for each candidate action by taking the softmax of the Q-values, the model sets *P*(*A*^1^|*S*^2^) = *m* and, for $A_{j}^{2}\neq A^{1}$,

$$P\left(A_{j}^{2}|S^{2}\right)=(1-m)\times \frac{P\left(A_{j}^{2}|S^{2}\right)}{1-P\left(A^{1}|S^{2}\right)}.$$

In total, the model has nine parameters: α^1^, β^1^, *f*^1^, γ^1^, α^2^, β^2^, *f*^2^, γ^2^, *m*.

## Results

### Overall performance

**Figure 2:**
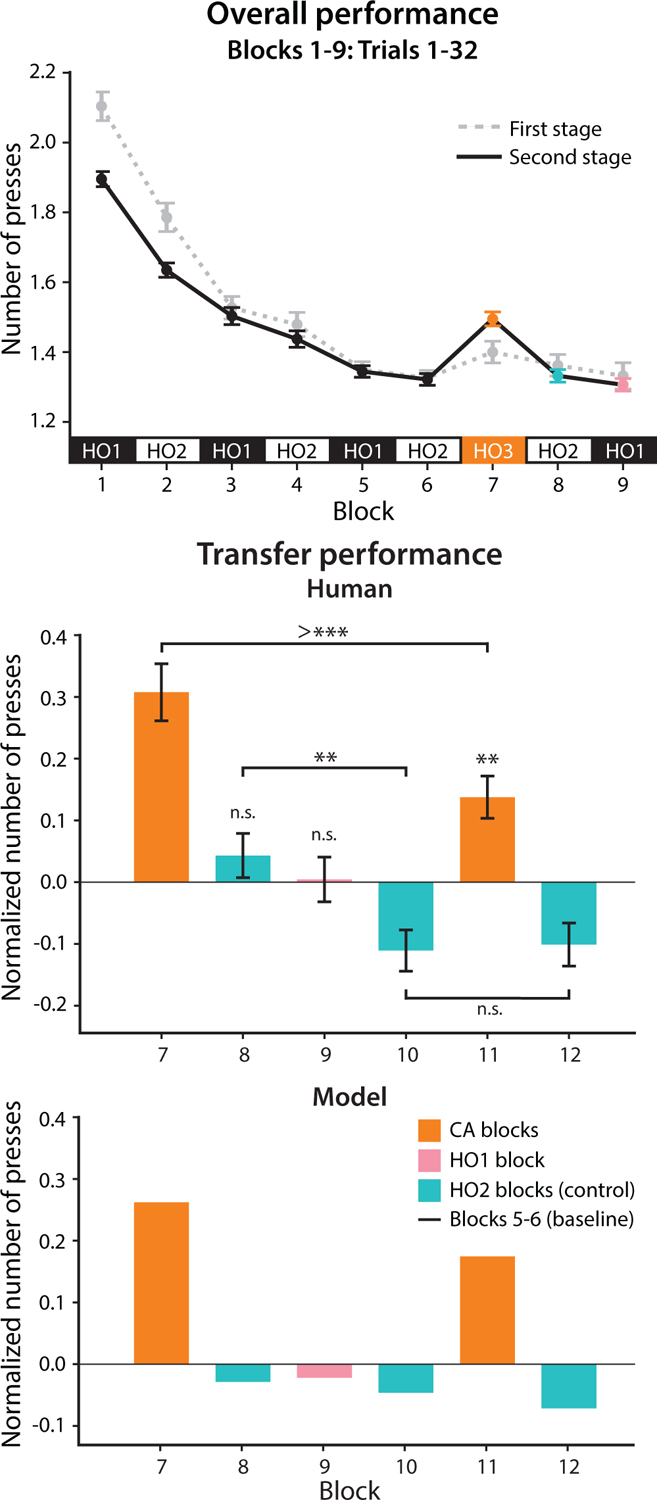
Learning curves of all participants (n=228) and transfer performance in Blocks 7–12 of participants who completed the 12-block versions of the task (n=129), with model simulations. Transfer performance was measured by the average number of presses in the first 10 trials of each block and normalized by baseline (see fig. 3 for separate plots of CA1 vs. CA2). We use n.s. to indicate *p* ≥ 0.05; * for *p* < 0.05; ** for *p* < 0.01; *** for *p* < 0.001; >*** for *p* < 0.0001. Error bars represent standard error of the mean.

The performance of all 228 participants in Learning and Test 1 (Fig. 2 top) replicated the results of Xia and Collins (2021). In the second stage of Learning, the average number of presses steadily decreased from 1.90 to around 1.32, which is substantially better than chance performance of 2.5. This suggests that participants effectively learned strategies to succeed in the task by creating and utilizing hierarchical options under HO_1_ and HO_2_. This was confirmed by analysis of early performance in Blocks 5–6 (data not shown). In Test 1, performance worsened (0.30 more presses than baseline in the first 10 trials, *p* < 0.0001), indicating negative transfer of HO_1_.

When retested on the originally learned HOs in Posttranfer 1, participants’ performance did not drop from baseline (p>0.05). In other words, the negative transfer in Test 1 did not interfere with participants’ performance on previously learned options. These results strongly suggest that in Test 1, a new set of options (denoted HO_3_) were created and credited, and these options did not overwrite the existing options (e.g., HO_1_ and HO_2_).

### Transfer performance

Next, we analyzed the transfer performance of participants who completed the 12-block versions of the task (n=129) in the Test and Post-test phases (Fig. 2 middle). Performance in Test 2 worsened from baseline (0.14 more presses in Test 2, p=0.0005), suggesting that the behavioral effects of negative transfer persisted after Test 1. In addition, performance in Test 2 was significantly better than in Test 1 (0.16 less presses in Test 2, p<0.0001). In the control HO_2_ blocks (even-numbered blocks), human performance stayed at baseline level in Block 8 (p>0.05), improved in Block 10 (0.15 less presses than in Block 8, p=0.0003), and was maintained in Block 12 (p>0.05 for Blocks 10 vs. 12). Using first press accuracy instead of number of presses to describe performance led to qualitatively identical results.

### Modeling

Using the Option Model, we simulated data for 2000 participants (Fig. 2 bottom). Because the model’s likelihood is intractable (Xia & Collins, 2021), parameters were not fitted to the data, but fixed manually (α^1^ = 0.2, β^1^ = 2, *f*^1^ = 0.001, γ^1^ = 5, α^2^ = 0.45, β^2^ = 6, *f*^2^ = 0.0001, γ^2^ = 20, and *m* = 0.05) to match experimental learning curves and show that the model can capture observed qualitative patterns. The model’s transfer performance was comparable to that of human participants: in Test 1, the number of presses increased from baseline by 0.26, similar to an increase of 0.30 presses in human participants; in Post-test 1, the model did not perform worse than baseline (0.03 and 0.02 decreased presses in Blocks 8 and 9, respectively). During and after Test 2, it showed similar patterns of performance to human participants. Effects of negative transfer persisted in Test 2 with increased presses. Moreover, performance improved in Test 2 from Test 1, as well as in Post-test 2 from Post-test 1.

### Behavioral effects of contextual similarity

**Figure 3:**
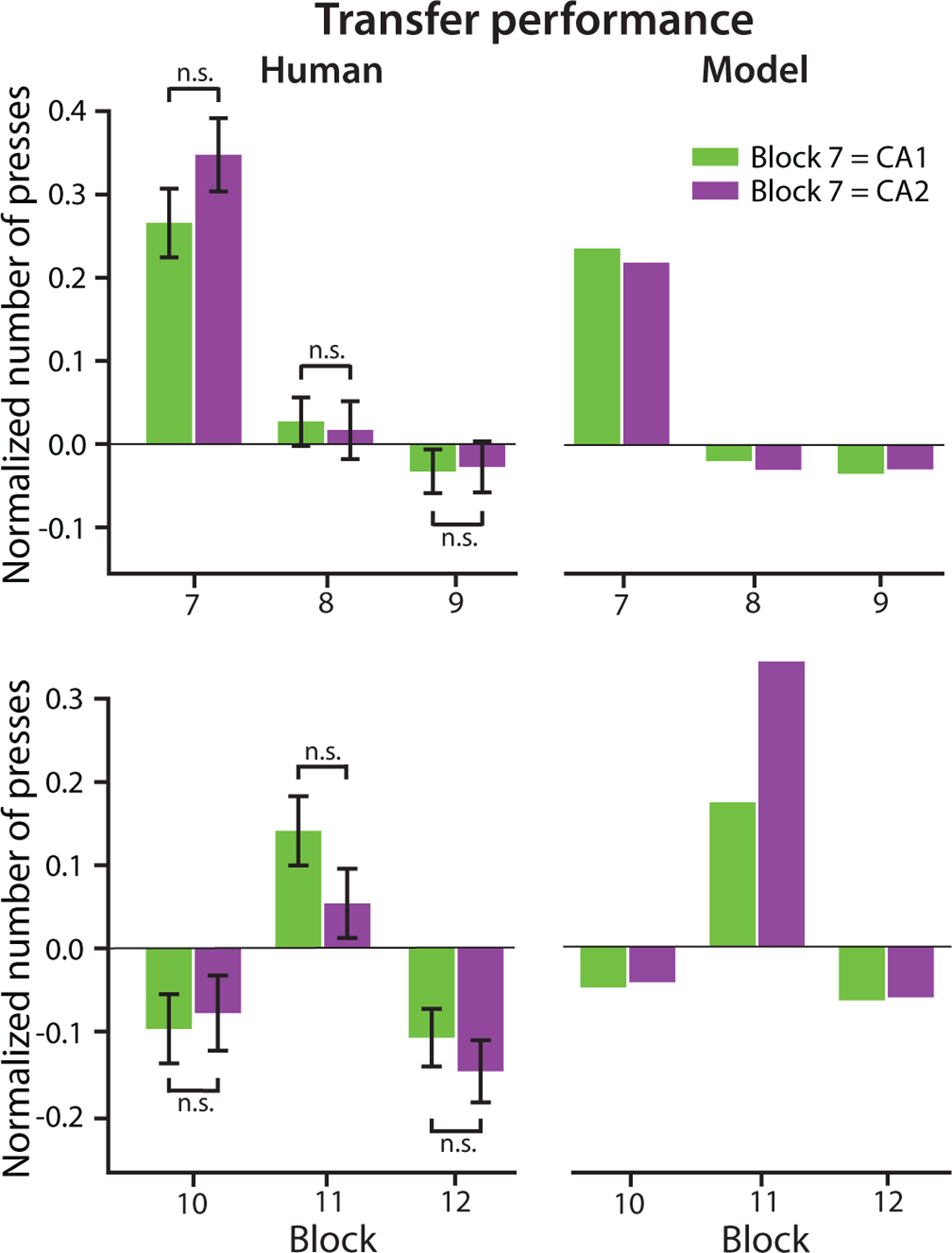
Comparison of transfer performance in the second stage of Blocks 7–12 (Test 1, Post-test 1, Test 2, and Post-test 2) between the CA1 and CA2 design of Test 1 in human participants and the Option Model.

The previous analyses collapsed over CA1 and CA2 in Test 1, which were contexts with less and more potential negative transfer. To test if the similarity between new and old contexts affected credit assignment in option transfer, we further compared transfer performance between CA1 and CA2 for human participants and the Option Model (Fig. 3). During and after Test 1, participants were divided into two groups based on the design of Test 1: CA1 (n=124) and CA2 (n=104). Similarly, the analysis on Test 2 and Post-test 2 included participants who completed the 12-block versions (n=67 for CA1 and n=62 for CA2) of the task. We found no effect of group on any metric of transfer performance in the second stage (Fig. 3 left). The option model could capture this pattern adequately for Test 1 and Post-test 1 (Fig. 3 top right). However, it predicted qualitatively more presses in Test 2 following CA2 than CA1 as Test 1, different than what we observed in participants (Fig. 3 bottom).

### Option learning of the model

**Figure 4:**
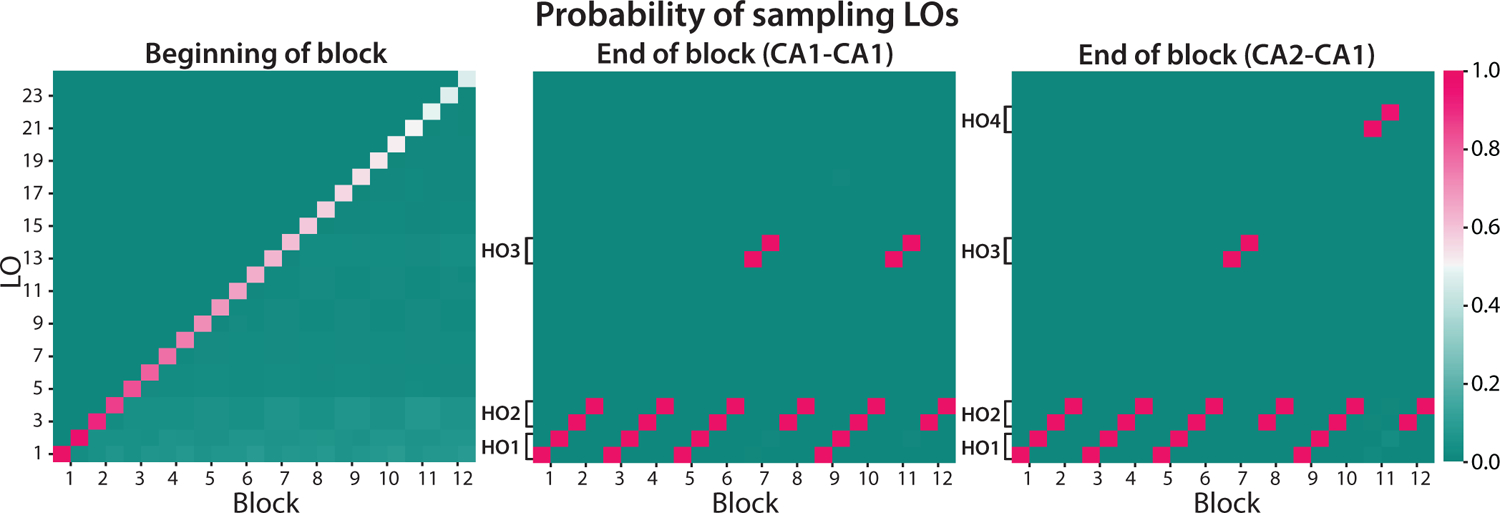
The probability of the model choosing different LOs in each second-stage context. X-axis: the block context defined by the identities of the block and first-stage stimulus. Y-axis: the LOs numbered based on the order by which they were created (two LOs were created at the beginning of each block, corresponding to the two distinct contexts per block).

To provide a clearer picture of which options the model learned and used, we illustrate the probability $P_{\text{MO}}^{2}$ of the model choosing each LO in the second stage of each block context over 1000 simulations (Fig. 4). In the beginning of each context, a new LO was always created with a probability of $P^{2}\left({\text{LO}}_{i}|c_{n+1}^{2}\right)=\frac{\gamma^{2}}{\gamma^{2}+Z^{2}}$, where $Z^{2}=\sum_{i=1}^{n}\sum_{k=1}^{n}P^{2}\left({\text{LO}}_{i}|c_{k}^{2}\right)$ increased with the number of contexts encountered. Thus, the probability of a new LO being sampled at the beginning of a block decreased as the task progressed. At the end of each block, the model had learned to exploit LOs that worked best for the current block contexts. In simulations where Test 1 was CA1, the model learned to use LO_1_ and LO_2_ in HO_1_ blocks, LO_3_ and LO_4_ in HO_2_ blocks, and LO_13_ and LO_14_ in CA1 blocks. Simulations where Test 1 was CA2 showed similar results, except that in Test 2, the CA block different from Test 1, the model exploited LO_21_ and LO_22_ rather than the same LOs it learned to utilize in Test 1.

## Discussion

The experimental data summarized above provided behavioral evidence for addressing both research questions we asked: in a new context where some contingencies have changed, do participants create new options or overwrite existing ones? Does the similarity of the new options to old options affect how they are credited?

We observed that participants successfully learned the updated hierarchical contingencies in Test 1, in which they outperformed chance by a large margin. Since performance improved back to baseline level when old contingencies were retested in Post-test 1, this strongly suggests that the old options learned in Learning were not interfered by the negative transfer in Test 1. Therefore, we argue that new options were reliably created in Test 1 and existing ones were not overwritten.

Based on our results (Fig. 4 top left), participants’ ability to assign credit to a new set of options (rather than a similar, old one) for the contingencies learned in Test 1 was not affected by how similar the new options were to existing options. Particularly, human transfer performance in Test 1 and Post-test 1 did not differ between CA1 and CA2 participants, which implies that credit assignment did not depend on the amount of negative transfer in the new options. This indicates very efficient credit assignment to a new option, avoiding interference with previously learned policies, even when they were very similar.

Surprisingly, we saw no benefits in Test 2 to participants who had a chance to transfer the newly learned Test 1 option (CA1 in Test 1) compared to those who needed to create a second new option (CA2 in Test 1). This might show that although participants created and assigned credit to a new option in Test 1, they did not consolidate it sufficiently to enable future transfer. As a result, the overall increase in performance from Test 1 to Test 2 was unlikely to reflect option transfer. Instead, it might indicate meta-learning of the task structure: for example, participants might have learned from Test 1 that block design could change from HO_1_ and HO_2_, and thus increased their prior probability over new options.

Overall, the Option Model captured human behavior well in learning, utilizing, transferring, and crediting options, despite its inaccurate prediction that transfer performance in Test 2 would be worse in CA2 participants than CA1. This discrepancy between the two simulated groups was due to the increasing difficulty to reject existing options and exploit new ones as more contexts had been encountered. On the algorithmic level, the fixed concentration parameter γ^2^ caused the probability of sampling newly created options to decrease over the course of the experiment (Fig. 4 left). Thus, when encountering a context with new contingencies later in the task, the agent spent more key presses learning to utilize new options, which led to worse performance in Test 2 when the context was new than if it was not.

This divergence in human and model behavior stemmed from a key limitation of the Option Model: it did not account for the meta-learning of humans. Unlike human participants who learned both the task structure and contingencies from scratch, the model was pre-programmed with metaknowledge of the task. Therefore, when meta-learning dominated learning, the model might fail to predict human behavior. In our experiment, human participants likely learned in Test 1 that the block design could change from the previous pattern, and thus adapted more quickly to such a change in Test 2. On the other hand, the model’s sampling probability of a newly created option was independent from how much the block design changed. Though the Option Model emulated some meta-learned behavior (e.g., the *m* parameter modeled a low probability of pressing the first-stage answer key in the second stage), it could not explain the process of meta-learning (e.g., how the hierarchies of the contingencies were learned). To improve the Option Model, future work could seek to better understand the meta-learning in this task with more nuanced data analysis or new experiments to tease apart the two types of learning.

Most importantly, our work corroborates and extends the theory that humans create, compose, and transfer hierarchical options by providing evidence for how new and old options are credited during transfer (Xia & Collins, 2021). However, some crucial questions remain challenging to answer with the current evidence, such as how option hierarchies are constructed and how well our simplified contexts and rules translate into real-world option learning. Eventually, we hope the options framework could help us understand humans’ exceptional ability to adapt to new contexts and transfer past knowledge to solve novel problems.

## Figures and Tables

**Table 1:** Dataset composition

Version	n (total)	% online	Test 1	Test 2
9-block CA1	57	61%	CA1	-
9-block CA2	42	62%	CA2	-
12-block CA1	67	76%	CA1	CA1
12-block CA2	62	74%	CA2	CA1
